# The Dynamics of Speed Selection and Psycho-Physiological Load during a Mountain Ultramarathon

**DOI:** 10.1371/journal.pone.0145482

**Published:** 2015-12-21

**Authors:** Hugo A. Kerhervé, Guillaume Y. Millet, Colin Solomon

**Affiliations:** 1 School of Health and Sport Sciences, University of the Sunshine Coast, Sippy Downs, Australia; 2 Laboratoire de Physiologie de l’Exercice, EA-4338, Université Savoie Mont Blanc, Le Bourget-du-Lac, France; 3 Human Performance Laboratory, University of Calgary, Calgary, Canada; 4 Laboratoire de Physiologie de l’Exercice, Université de Lyon, F–42023, Saint–Etienne, France; University of the Balearic Islands, SPAIN

## Abstract

**Background:**

Exercise intensity during ultramarathons (UM) is expected to be regulated as a result of the development of psycho-physiological strain and in anticipation of perceived difficulties (duration, topography). The aim of this study was to investigate the dynamics of speed, heart rate and perceived exertion during a long trail UM in a mountainous setting.

**Methods:**

Fifteen participants were recruited from competitors in a 106 km trail mountain UM with a total elevation gain and loss of 5870 m. Speed and gradient, heart rate (HR) and ratings of perceived exertion (dissociated between the general [RPE_GEN_] and knee extensor fatigue [RPE_KE_] and collected using a voice recorder) were measured during the UM. Self-selected speed at three gradients (level, negative, positive), HR, RPE_GEN_ and RPE_KE_ were determined for each 10% section of total event duration (TED).

**Results:**

The participants completed the event in 18.3 ± 3.0 h, for a total calculated distance of 105.6 ± 1.8 km. Speed at all gradients decreased, and HR at all gradients significantly decreased from 10% to 70%, 80% and 90%, but not 100% of TED. RPE_GEN_ and RPE_KE_ increased throughout the event. Speed increased from 90% to 100% of TED at all gradients. Average speed was significantly correlated with total time stopped (r = -.772; p = .001; 95% confidence interval [CI] = -1.15, -0.39) and the magnitude of speed loss (r = .540; p = .038; 95% CI = -1.04, -0.03), but not with the variability of speed (r = -.475; p = .073; 95% CI = -1.00, 0.05).

**Conclusions:**

Participants in a mountain UM event combined positive pacing strategies (speed decreased until 70–90% of TED), an increased speed in the last 10% of the event, a decrease in HR at 70–90% of TED, and an increase in RPE_GEN_ and RPE_KE_ in the last 30% of the event. A greater speed loss and less total time stopped were the factors associated with increased total performance. These results could be explained by theoretical perspectives of a complex regulatory system modulating motor drive in anticipation of perceived difficulties such as elevation changes.

## Introduction

Exercise intensity during self-paced events is regulated by a complex protective system integrating instantaneous somatosensory feed-back and anticipatory mechanisms in order to maintain homeostasis and prevent from catastrophic failure [1–3]. As in shorter duration, self-paced exercises [2, 4–8], the dynamics of exercise intensity during ultramarathons (UM) are expected to be regulated as a result of the development of psycho-physiological strain and in anticipation of perceived difficulties (duration, topography) [1]. Despite being sensitive to environmental factors such as gradient and wind [9], speed is commonly used as an indicator of exercise intensity in the study of athletic performance. Speed is predicted to decrease throughout UM events [10], and systematic descriptions of pacing during field UM events have been used to indicate that speed decreases overall and becomes more variable as a function of increased finishing times [11–14].

UM events performed on trails and in mountainous settings include changes in elevation, surface, obstacles, altitude, remoteness, and adverse atmospheric conditions, which could alter the dynamics of pacing, compared to flat events. For instance, significant peripheral fatigue (low-frequency fatigue, indicative of the failure of excitation-contraction coupling typical of exercises involving intense eccentric and stretch-shortening contractions [15, 16]) was observed following a ~37 h mountain UM event [17], but not following a 24 h level, treadmill run [14]. Therefore, it is possible that the dynamics of speed during a mountain UM would differ compared to a level UM.

Some measures of psycho-physiological load, such as the ratings of perceived exertion (RPE) and heart rate (HR), are strong predictors of pacing during self-paced exercise [1, 18], and could assist in characterising the psycho-physiological load pertaining to a runner at various stages of an UM. HR is an indicator of the cumulative systemic physiological response to variations in physiological and psychological load, and is routinely used to monitor exercise intensity [19]. The RPE are scores of specifically-developed subjective scales [20] widely used as indicators of psycho-physiological strain [2], and have been proposed to be a primary variable involved in the selection of work rate [1, 2]. During a 68km UM lasting 9.8 ± 0.4 hr, HR decreased in the second half, and RPE increased throughout the event without reaching maximal values (15.4 ± 0.4) [21]. RPE was also found to increase and to have slightly higher values (14.1 ± 2.0, with a maximum under ~18) throughout a 73 km mountain UM lasting 11.5 ± 0.5 hr [22]. More recently, near-maximal RPE values (19.5 ± 1.5) were reported at the end of a 54 km mountain UM despite a moderate exercise intensity as evidenced by the relatively low average speed (3.83 km ∙ h^−1^), and HR (111.7 ± 5.9 bpm) [23]. However, no description of psycho-physiological load and pacing has been performed in long, mountain UM events.

Therefore, the aim of this study was to investigate the dynamics of speed, HR and perceived exertion during a long trail UM in a mountainous setting. We hypothesised that a progressive decrease in speed (positive pacing) and increased variability would be observed in participants. Based on shorter duration events [11, 24], we also hypothesised that changes in speed would be correlated to changes in gradient, and would exhibit different dynamics at level gradients compared to positive and negative gradients. In association with these changes, we hypothesised that HR would decrease (reverse HR drift), and that RPE would increase throughout the event. Together, these findings would indicate that pacing is regulated not only as a consequence of the development of fatigue (simultaneous decrease in speed and HR, [25]), but also in an anticipatory manner to prevent from reaching high levels of exertion prematurely.

## Methods

### Ethics statement and participants

Ethical approval for the study was granted by the research ethics committee of the University of the Sunshine Coast (project code S/12/432). The participants were recruited from experienced runners registered to compete in a ~167 km UM running event with ~10,000 m of elevation gain and loss, and held in Chamonix, France (The North Face^®^ Ultra-Trail du Mont-Blanc, UTMB). Nineteen male participants provided written informed consent and were initially included in the study, fifteen of which completed the entire event and constitute the study group (age: 43 ± 10 yr, height: 1.78 ± 0.60 m, weight: 74 ± 8 kg).

### Study procedures

The participants were individually familiarised with the following study procedures in the 3 days preceding the event. Due to adverse meteorological conditions, the course was shortened to ~106 km and the elevation gain and loss reduced to ~6,000 m on race day.

#### Measures of distance, speed and gradient

The participants were equipped with a Non-differential Global Positioning System (GPS_ND_) device (BT-Q1000, Qstarz International, Taiwan) secured on top of their clothing or gear to record the distance, speed and elevation for the entire event. The GPS_ND_ data were retrieved using the proprietary software, and exported in columnar format for data analysis. The variables contained in the files were date and time (universal time constant, UTC), position (latitude, longitude, elevation) and speed (via Doppler shift).

From the successive geographical coordinates recorded, we calculated the distance between each data point using the Vincenty great-circle formulae [26], which are spherical trigonometry functions calculating the shortest distance between spatial coordinates at the surface of an ellipsoid (earth dimensions used were the WGS-84 GPS model of reference with equatorial radius ≈ 6,378.137 km, polar radius ≈ 6,356.752 314 245 km and flattening f≈1298.257223563). An automated calculation of the Vincenty formulae can be obtained from an internet-based utility (GPS Visualizer; www.gpsvisualizer.com), which we compared to our preliminary measures for 10 data sets and found to be in exact agreement (r = 1.00, p < .001). Therefore, we used the automated formulae as a simple and generalisable procedure to obtain point-to-point distances. Point-to-point speed was subsequently calculated using the ratio of the point-to-point distances and of the GPS epoch time (one data point every 5 s in the current study).

**Fig 1:**
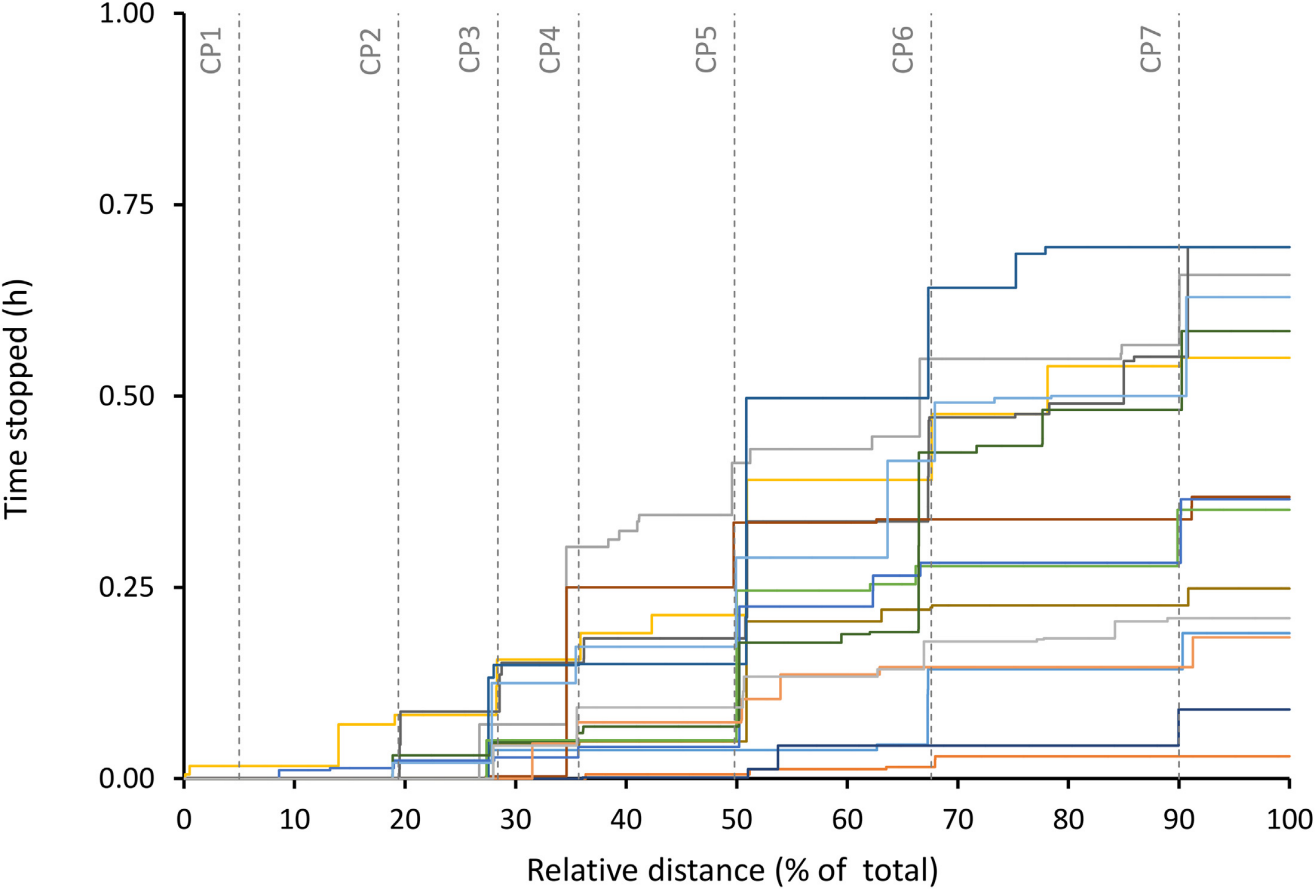
Total time stopped. Total time stopped for each participant, including the relative position of official event checkpoints. “CP” are official event checkpoints. CP 1 and 6: distance ~7 km (outbound) and ~69 km (inbound), altitude ~1015 m. CP 2: distance ~ 19 km, altitude ~815 m. CP 3 and 5: distance ~29 km (outbound) and ~52 km (inbound), elevation ~1160 m. CP 4: distance ~36.5 km, elevation ~1699 m. CP 7: distance ~93 km, elevation ~1263 m.

To reduce the effect of signal errors in the analysis, a two-step treatment procedure was then applied to the data. Preliminary calculations revealed that GPS_ND_ devices did not discriminate speeds slower than 1 km ∙ h^−1^ (0.28 m ∙ s^−1^ or 1.39 m in 5 s) based on the typical error in speed measured in a static position (drift, when a device will record speed values due to the non geo-synchronous nature of the constellation of satellites). For the high end of the speed range, it was considered that speeds higher than 20 km ∙ h^−1^ (5.56 m ∙ s^−1^ or 27.8 m in 5 s) were not expected during a long UM and were likely due to signal jamming (which occurs mainly when the signal from a satellite becomes too weak and forces the ground based receiver to pair to another satellite). These erroneous distance and speed data were first assigned a value of zero, and all speed values were then smoothed in order to further increase the signal-to-noise ratio. For smoothing, a 3, 9, and 15-pt weighted averages were graphically compared. The 9-pt weighted average was considered satisfactory as it provided a balanced sensitivity to individual observations of slow and high speeds. This two-step procedure facilitated the reduction of the effect of signal drift and jamming, which both artificially increase the distance and speed measured using GPS devices, while remaining sensitive to periods of null speed values (Fig 1).

GPS-based elevation is considered to be inaccurate [27] due to differences between the model of reference of the earth used for calculations and the actual shape of the earth, and therefore an independent source was sought in order to increase the quality of the elevation data. Due to the size of a typical file containing UM data at the relatively high recording rates of GPS devices (12 h of data recording at 5 s equals 8640 observations), a digital elevation model (DEM) was used in order to automate the treatment procedure. Elevation values were reconstructed from the geographical positions using a DEM (in this study, the NASA SRTM3) available from the same online utility (GPS Visualizer; gpsvisualizer.com). Data was smoothed using a 9-pt weighted average. The gradient between two consecutive data points was then calculated as the change in elevation divided by the horizontal distance between two points (the amount of vertical gain as a function of horizontal distance).

#### Measures of psycho-physiological load

HR was measured continuously using a chest strap and watch (RS800, RS800cx, RS400, or S810, Polar Electro, Kempele, Finland). Each watch was set to record one data point every 15 s in order to optimise battery life and memory.

RPE scores were recorded using a portable voice recorder (ICD PX312, Sony, Tokyo, Japan). We instructed the participants to record the time of observation, a general (RPE_GEN_) and a local (muscular) RPE focused on the sensation of fatigue or pain of the knee extensor muscles and excluding any psychological/psychic contribution to exertion (RPE_KE_) using Borg’s 10 point category-ratio scale (CR-10) that the subjects carried over the entire race.

### Variables and statistical analyses

**Fig 2:**
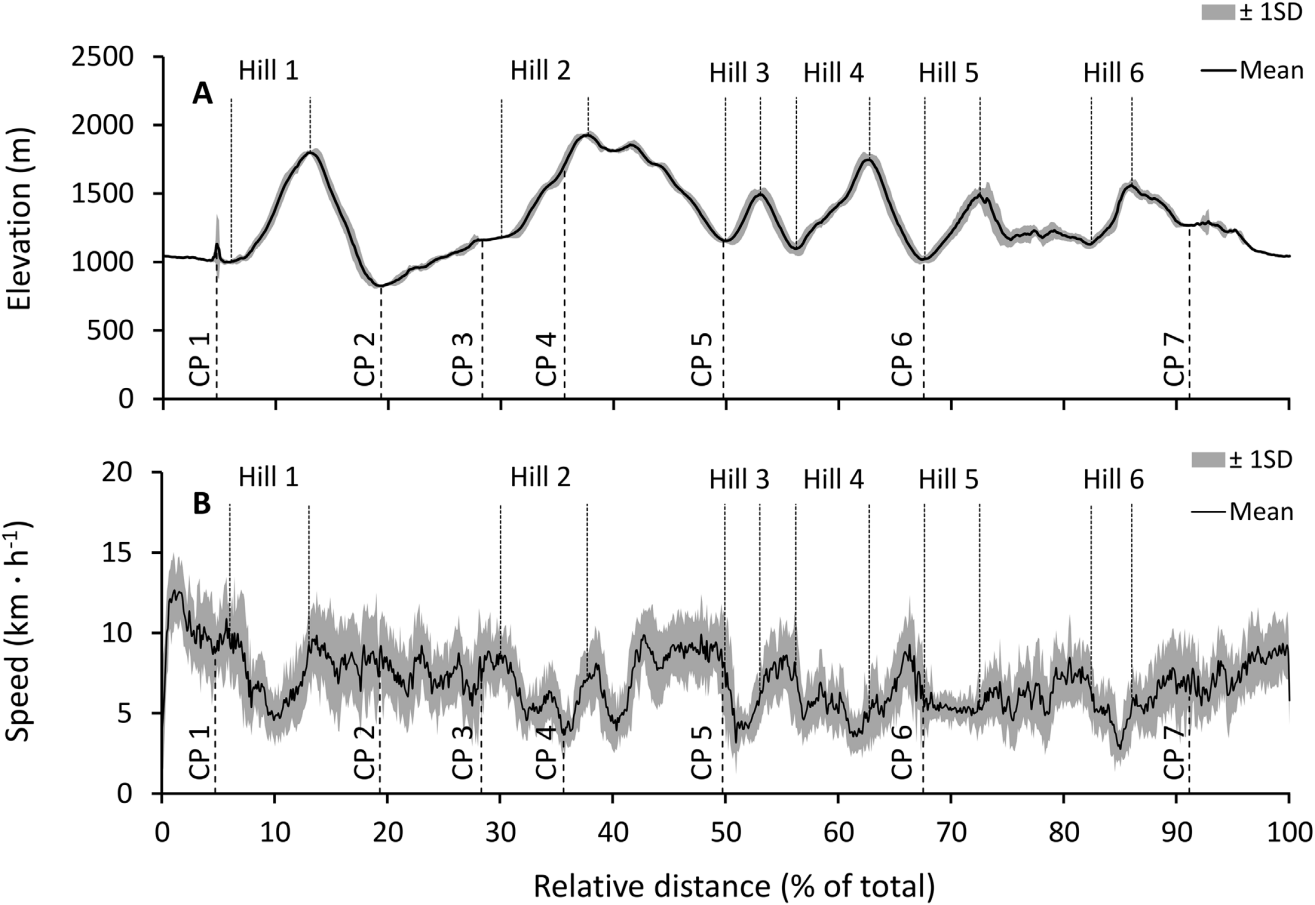
UTMB course outline and elevation profile. (A) Mean (±SD) group elevation data from the Digital Elevation Model values associated with measures of geographical positions, and (B) group speed data associated with measures of geographical positions (CP, refer to legend of Fig 1 for description).

**Fig 3:**
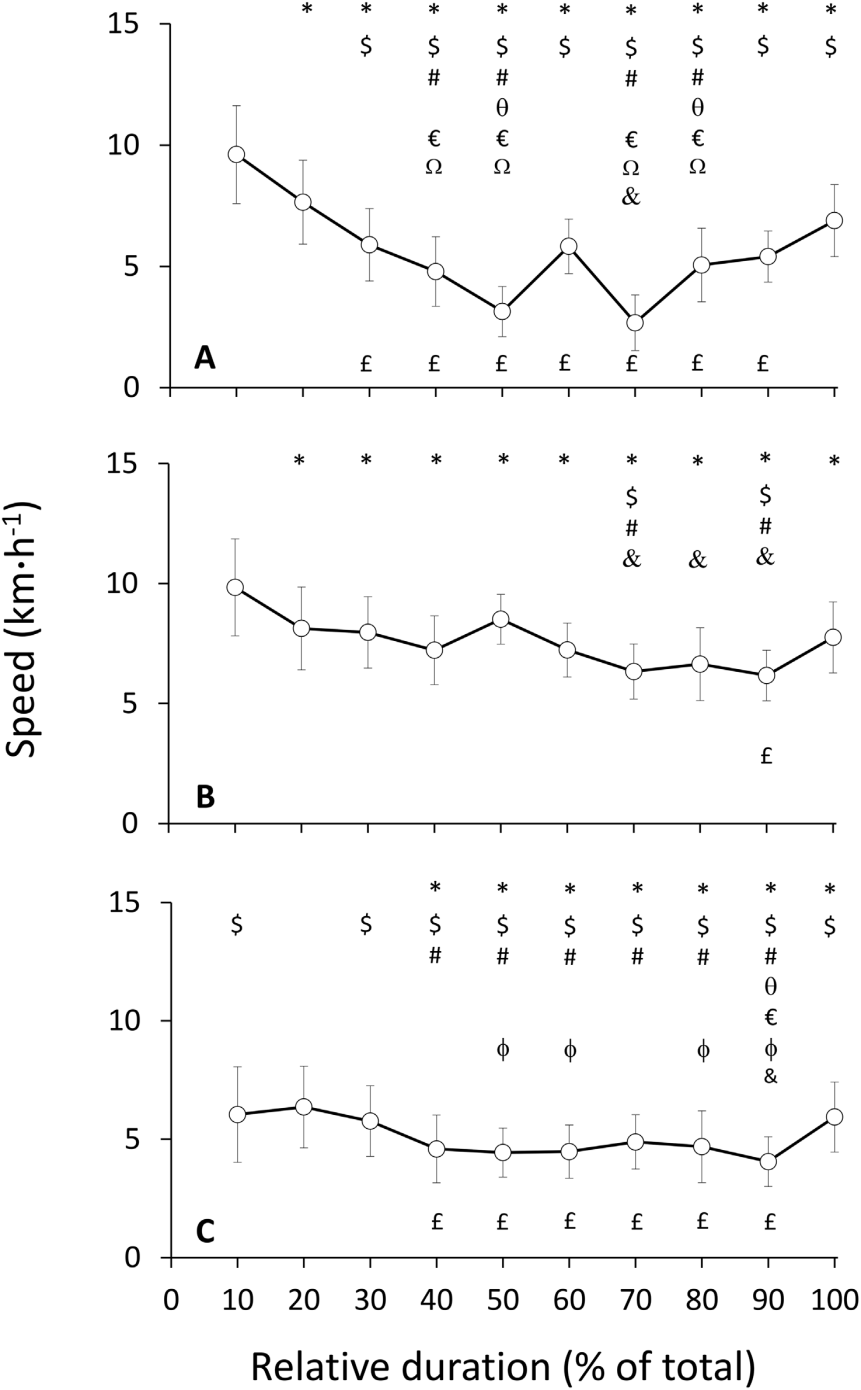
Dynamics of speed. Mean (±SD) group speed as a function of event duration in (A) level, (B) negative (C) and positive gradients, respectively. Symbols denote significant differences to (*) 10%, ($) 20%, (#) 30%, (θ) 40%, (&) 50%, (€) 60%, (ϕ) 70%, (Ω) 90% and (£) 100% of total event duration, at p < .05.

**Fig 4:**
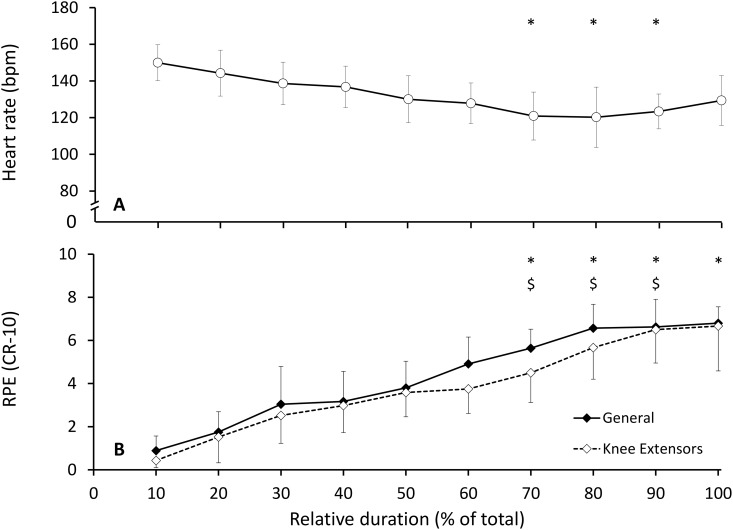
Dynamics of psycho-physiological load. (A) Mean (±SD) group heart rate (HR) as a function of total event duration, and (B) general and muscular (knee extensors) ratings of perceived exertion (RPE) as a function of total event duration. Symbols denote significant differences to (*) 10% and ($) 20%, at p < .05. Bpm: beats per minute. CR-10: 10-point category-ratio Borg scale.

We reported all variables as a function of total event distance (Figs 1 and 2) or duration (Figs 3 and 4) in order to represent all participants on a comparable scale (where 100% represents the distance or duration at event completion for every participant). In order to ensure sufficient data was used at each stage, data for each dependent variable was computed for every 10% section of the total duration of the event. All statistical analyses were performed using SPSS (version 21, IBM Corporation, Armonk NY, USA). Data are reported as mean ± SD, and the level of significance was set at p < .05.

#### Dynamics of exercise intensity

We determined the relationship between the variations of speed and changes in elevation for the entire event using a quadratic regression of individual speed and gradient. After confirming the assumption of the equality of variances were met, the effect of exercise duration on speed at each gradient (level, negative and positive inclines) HR, RPE_GEN_ and RPE_KE_ were determined using a multivariate ANOVA (MANOVA). Post-hoc one-way, repeated measures ANOVAs with a Fisher’s LSD post-hoc test were used in order to locate the differences in means for speed at all gradients. Due to incomplete data sets, the dynamics of HR, RPE_GEN_ and RPE_KE_ were assessed using a one-way ANOVA on ranks (Kruskal-Wallis test) with a Student-Newman-Keuls post-hoc test.

As HR does not adjust to exercise intensity instantaneously, it is not possible to treat HR data in the same way as speed. Instead, we investigated the dynamics of exercise intensity using sections of sustained uphill running, in order to maximise the contribution of metabolic work compared to passive energy recovery (since the ability to perform eccentric contractions decreases with the development of peripheral fatigue, refer to [15, 16]) in total work rate [28]. We identified 6 sections of sustained uphill combining at least 300 m of vertical gain at a 10% average gradient (refer to Fig 2; the section from CP2 to CP3 was only 4.9% gradient and was not included in the analysis). The effect of hill order (1 to 6) on HR was assessed using a repeated-measures, one-way ANOVA and a Bonferroni post-hoc test.

However, during uphill running, overground speed is a less relevant metrics than the amount of vertical gain (in m ∙ h^−1^) to characterise exercise intensity, which is also dependent on the gradient of the slope [28]. Therefore, to determine whether any drift in HR existed independent of exercise intensity, we used a 1-factor principal component analysis to reduce the dimension of these three variables (termed SVG for speed, vertical gain, gradient). After confirming the assumptions of normality using a Shapiro-Wilk test, the relationship between average HR and SVG in the 6 main ascents was assessed using Pearson’s product-moment correlation. We further tested the effect of hill order (1–6) on HR using a repeated-measures one-way ANCOVA, and a Bonferroni post-hoc test, using SVG as a covariate of HR.

#### Factors of performance

The relationship between final performance (using the individual average speed, as it allows comparison of various UM distances) and 1) the variability of speed (using the coefficient of variation of point-to-point speed values), 2) the magnitude of speed loss (using the slope of the linear regression of speed over the entire event) and 3) the total time stopped (assumed to correspond to resting, eating, clothing and gear change, toilet, other) were tested using Pearson’s product-moment correlation after confirming assumptions of normality were met (Shapiro-Wilk test). The 95% confidence intervals (CI) of the correlations were calculated using the unstandardised beta-weights of the linear regression of the Z-scores of each variable.

## Results

The following data sets were retrieved: 15 complete GPS traces, 9 HR data sets with at least 80% of event data (due to equipment issues and loss of signal), and 6 RPE data sets with at least 80% of data. The average distance for the event, calculated using filtered point-to-point orthodrome, was 105.6 ± 1.8 km (range: 103.0–107.5 km), and the total elevation gain and loss was 5871 ± 239 m. The 15 participants completed the event in 18.3 ± 3.0 h (range: 13.8–23.9 h) at an average speed of 5.88 ± 0.9 km ∙ h^−1^ (range: 4.58–7.58 km ∙ h^−1^) and an average HR of 132 ± 10 bpm (range: 112–146 bpm). The mean group elevation and speed profiles are represented in Fig 2. There was a significant quadratic correlation between point-to-point speed and elevation changes (linear factors model: r = .49, R^2^ = .24, F-linear = 316.76, p < .001; quadratic factors model: r = .52, R^2^ = .27, F-change = 40.99, p < .001; Total factors: F-total = 185.22, p < .001).

### Dynamics of exercise intensity

The changes in speed as a function of total event duration are presented in Fig 3 (panels A, B and C for level, negative and positive gradients, respectively). Positive pacing was observed on level (speed loss: -2.91 ± 2.15%), negative (-2.61 ± 0.92%) and positive gradients (-1.31 ± 0.84%). The MANOVA indicated a difference in speed between the speed at level gradient, and at the negative and positive gradients (p < .001). Speed was not significantly different between the negative and positive gradients (p = .10). Post-hoc ANOVAs indicated that speed decreased from 10% to all sections up to 70% of total duration at level inclines (except 60%, where it significantly increased compared to 40% and 50%), and that speed increased at 90% compared to 40%, 50%, 70%, 80%, and increased at 100% compared to all sections between 30% and 90% (Fig 3A). For negative gradients, speed decreased from 10% to all other sections, and from 20% and 30% to 70% and 90%. Speed then increased between 90% and 100% (Fig 3B). For positive gradients, speed decreased at all sections until 80% of event duration except 60%. Speed increased at 100% compared to all observations between 40% and 90% (Fig 3C).

Mean group HR averaged 132.6 ± 13.6 bpm, and decreased -34.2 ± 17.2 bpm over the UM. HR decreased from 10% to 70%, 80% and 90%, but not 100% (Fig 4A). Mean group RPE_GEN_ and RPE_KE_ averaged 4.3 ± 1.1 and 3.8 ± 1.3, respectively, and increased 6.9 ± 1.4 and 6.9 ± 2.2, respectively during the event (Fig 4B). Mean group RPE_GEN_ increased significantly from 10% to 70%, 80%, 90% and 100%, and from 20% to 70%, 80% and 90% (Fig 4B). Mean group RPE_KE_ increased significantly from 10% to 70%, 80%, 90% and 100% (Fig 4B). Mean group RPE_GEN_ and RPE_KE_ were significantly positively correlated (r = .980, p < .001).

There was no significant change in HR as a function of time in the 6 main climbs, as evidenced in the ANOVA (using HR alone) as well as in the ANCOVA (using HR and SVG as covariates). HR was significantly positively correlated with SVG on all uphill sections (r = .663, p < .001).

### Factors of performance

Performance (average speed) was negatively correlated with total time stopped (r = -.772, p = .001; 95% CI = -1.15, -0.39) and positively correlated with the magnitude of speed loss (r = .540, p = .038; 95% CI = -1.04, -0.03) but not with the variability of speed (r = -.475, p = .073; 95% CI = -1.00, 0.05).

## Discussion

There were three main findings in this study: (i) speed decreased overall at all inclines during the event (positive pacing), but increased significantly in the last section at all inclines; (ii) faster participants stopped less and decreased their speed more than slower participants throughout the event; and (iii) the measures of psycho-physiological load indicated that despite evidence of a reverse HR drift and increased RPE throughout the event, HR in sustained climbs did not change, and maximal RPE values were relatively low, suggesting that participants actively regulated (paced) their physiological and psychological load to complete the event and avoid premature exhaustion.

During self-paced running exercise, the optimal locomotor speed is adjusted as a function of environmental factors [10]. One of the main factors influencing speed selection is gradient, where additional energy is required to run at high negative (to generate braking forces limiting downward acceleration) and positive (to elevate a runner’s mass against gravity) gradients compared to level or slightly negative gradients [28, 29]. The curvilinear relationship between locomotor speed and gradient measured in this study (indicating that speed varies directly as a function of gradient; Fig 2) had so far been assumed to exist [11] but not measured in UM events due to limitations in the ability to measure speed and gradient of individual participants. This relationship was measured despite the overall low speeds (especially at positive gradients) and positive pacing strategies characteristic of mountain UM events, which could both have affected the relationship.

The overall decrease in speed during the event indicates that the study participants used positive pacing strategies (progressively slowing down), in agreement with previous research findings specific to UM running [10–13, 25, 30]. Speed losses on level gradients were more pronounced (the slope of the linear regression was greater, and the MANOVA indicated that significant differences existed with both negative and positive gradients) and occurred at an earlier point in the event (reaching a minimum at 70% of event duration) compared to both negative and positive gradients (minimum at 90% of event duration). The increase in speed at 60% of total event duration on level gradients could not be explained using the data we collected. This section corresponded to a section of the course in the main valley of Chamonix, and therefore we hypothesise that the terrain and surface were conducive for running (in contrast to walking), and that the course was accessible by spectators and crew members (which could have provided support and motivation). The existence of an increase in speed at the end of the event at all inclines in the current study is unique compared to other studies of long UM on level ground [13]. Two combined factors could, in part, explain the presence of an increase in speed in the last section: first, the mainly descending profile at the end of the event (after hill 6, refer to Fig 2), and second, a phenomenon termed cardio-pulmonary [7] or speed reserve [1] predicting the increase of exercise intensity at the end of self-paced exercises. In this mountain UM event, the marked elevation gain and loss may have favoured the use of conservative pacing strategies decreasing the risk of premature exhaustion in anticipation of difficulties, when compared to level or hilly UM events [1]. Together, these findings are indicative of mixed pacing strategies, which is a subset of the three main types of pacing associating positive pacing for the main part of the event and an increase in speed for the final section of the event.

The significant relationship between performance and speed loss in this project contrasts with findings in other UM studies [11, 13], where participants with a higher performance level had greater speed losses. Future studies are required to further investigate this unexpected result, which could potentially originate from faster participants pacing their race less conservatively from the start aiming to decrease overall time, compared to slower participants, for whom finishing the event could have been the main goal. Future studies should investigate a priori pacing strategies, and performance goals and attitudes toward risk taking as a function of performance level. The inverse correlation between performance and time stopped was novel in UM running, and extends findings in an ultra-endurance cycling event [31] where faster athletes spent less time napping. While this result is expected in shorter duration events, it is commonly believed among participants of ultra-long duration events that a bout of passive rest can be beneficial to final performance. This finding indicates the marked differences in the physiological demands of an UM event as a function of performance level due to the differences in time spent on course, where passive rest may be a relatively important feature of pacing strategies for slower participants. Future studies are also required to determine whether a threshold exists as a function of performance level in longer (> 300 km) UM events.

We reported that HR decreased from 10% to 70%, 80% and 90%, but not 100% of total event duration. Although we could not determine the relationship between the dynamics of speed and HR at each gradient over the entire event (due to the relatively low accuracy of GPS_ND_ devices), it is likely that the variations of exercise intensity (speed) explained most of the observed changes in HR (reverse HR drift), as this well-described physiological response is typical of ultra-long duration exercise [1, 25]. Still, we reported that HR in sustained uphill sections distributed throughout the event (hills 1–6) did not change, including with the use of HR scaled for exercise intensity (using the factorial component SVG), which indicates that bouts of sustained uphill running may be regulated differentially given the high risk of exhaustion they present.

The dissociated RPE (RPE_GEN_ and RPE_KE_) had similar dynamics (the two measures of RPE increased from 10% to all sections between 70% and 100% of event duration), and were highly correlated, suggesting either (i) that the dynamics and magnitude of change of RPE_GEN_ and RPE_KE_ are similar throughout the course of an UM (which would indicate that the measure of one variable is sufficient), or (ii) that the two scales measured the same underlying construct (and therefore, that RPE may be a general, but not location-specific indicator of psycho-physiological load). Future research is required to investigate the relative contribution of the perceived exertion specific to a muscle group (knee extensors, plantar flexors) or physiological system (respiratory, gastric) to the general RPE. The role of pain will also need to be investigated as it may alter the perception of exertion and fatigue [1], and will be heightened following sustained downhill locomotion [32]. Further, although the highest values in RPE (both RPE_GEN_ and RPE_KE_) were recorded in the last section of the event, the maximal values were relatively low (6.3 and 6.7 group mean for RPE_GEN_ and RPE_KE_, respectively). The relatively low maximum values distinguishes it from other studies in shorter UM [23], and could contribute to identify the protective nature of fatigue in preventing the participants from attaining maximal values at the end of the event [1, 3]. As such, the combined results of the dynamics of pacing and psycho-physiological load may indicate that participants relied on relatively conservative pacing strategies and used a functional reserve [1, 7] permitting an increase in speed observed in the last section of the event. These findings are consistent with theoretical perspectives of a complex protective system regulating work rate based on the interaction of the instantaneous and anticipated psycho-physiological state of a participant, and of the environmental conditions in which the exercise is performed [1–3]. Still, some questions remain regarding the regulation of speed at different gradients, since some of our results (the pacing on level gradients differed to both downhill and uphill gradients) were not expected. Previous research hypothesised that the changes in stride patterns were altered differentially following a level [33] and a mountain UM [32] due to the increased reliance on eccentric contractions typical of downhill running. Recently, Vernillo and colleagues [34] measured an increase in the energy cost of running specifically at mild downhill (-5%) gradients but not on level or uphill (5%) gradients as a function of the development of fatigue in an UM event. Therefore, further studies are required to investigate the simultaneous variations of pacing and psycho-physiological load as a function of gradient during an UM event with a greater resolution.

### Limitations

In this study, the main limitation was related to the resolution of measurements made using non-differential GPS devices. We optimised the calculations to be able to define broad categories of total distance and duration (10% sections) and gradients (negative [-100 to -2.5%], level [-2.5 to 2.5%], positive [2.5 to 100%]). Still, the temporal resolution of observations (10% of event duration) was comparable to other UM studies [13], and was selected as a robust approach ensuring a sufficient number of observations was available for each variable. Unforeseen issues with recording equipment reduced the numbers of data sets in HR, and therefore limited the findings as we were unable to establish the relationship between HR and pacing at each stage. In future studies, the use of GPS devices with higher temporal and spatial resolution could also lead to the development of various indices of running performances in conditions of trail running, such as the rate of ascent as a function of gradient which would be useful for athletes and coaches, and in scientific research for the analysis of performance.

## Conclusion

During a mountain UM, speed decreased over the first 90% of the event and at all gradients, and speed increased in the last 10% section of the event. A greater speed loss and less total time stopped were the factors associated with increased total performance. HR decreased overall, but remained constant in the main ascents of the race, indicating the potential effect of conservative pacing strategies to avoid premature exertion. Perceived exertion increased throughout the event, but without reaching maximal values. These observations are supported by theoretical perspectives of a complex protective system regulating motor drive in anticipation of remaining exercise duration and changes in elevation.
